# Initiation of dolutegravir vs. efavirenz on 12- and 24-month retention and viral suppression: a target trial emulation

**DOI:** 10.1080/23744235.2025.2557628

**Published:** 2025-09-22

**Authors:** Amy Zheng, Alana T. Brennan, Ross Greener, Emma M. Kileel, Jacob Bor, Willem D. F. Venter, Eleanor J. Murray, Pedro T. Pisa, Bridgette Goeieman, Matthew P. Fox, Mhairi Maskew

**Affiliations:** a Department of Epidemiology, Boston University School of Public Health, Boston, MA, USAs; b Department of Global Health, Boston University School of Public Health, Boston, MA, USA; c Faculty of Health Sciences, Health Economics and Epidemiology Research Office, University of the Witwatersrand, Johannesburg, South Africa; d Department of Internal Medicine, Faculty of Health Sciences, University of the Witwatersrand, Johannesburg, South Africa; e Faculty of Health Sciences, Wits Ezintsha, University of the Witwatersrand, Johannesburg, South Africa; f Department of Public Health Medicine, School of Health Systems and Public Health, Faculty of Health Sciences, University of Pretoria, Pretoria, South Africa; g Right to Care, Johannesburg, South Africa

**Keywords:** South Africa, dolutegravir, efavirenz, retention, viral suppression

## Abstract

**Background:**

South Africa’s antiretroviral therapy (ART) treatment guidelines in 2019 were revised to use dolutegravir as part of first-line ART instead of efavirenz due to recommendations from the World Health Organization and findings from clinical trials indicating noninferior efficacy and reduced side effects. Utilizing the target trial framework, we estimated the effect of initiating a dolutegravir-based regimen compared to an efavirenz-based regimen among treatment-naïve people living with HIV initiating treatment in Johannesburg, South Africa from 2019 to 2022 on retention and viral suppression.

**Methods:**

We used linear regression to estimate causal risk differences on 12- and 24-month retention and viral suppression. Characteristics of those who initiated dolutegravir vs. efavirenz were balanced through inverse probability of treatment weighting. The covariates included: natal sex, age, year of initiation, education level, employment status, tuberculosis, WHO stage, smoking and alcohol use.

**Results:**

Of the 2930 individuals initiating ART, 1847 initiated a dolutegravir-based regimen and 1083 initiated an efavirenz-based regimen. The median age was 45.1 years (IQR: 37.1, 53.0). Initiation of dolutegravir was associated with a 5-percentage point increase (95% confidence interval (CI): −0.02, 0.11) in retention and 4-percentage point increase (95% CI: −0.06, 0.16) in viral suppression at 12 months. At 24 months, dolutegravir was associated with a 10-percentage point (95% CI: 0.03, 0.16) increase in retention and a 14-percentage point (95% CI: −0.02, 0.30) increase in viral suppression.

**Conclusions:**

Initiation of dolutegravir led to an appreciable increase in retention and viral suppression over 24 months when compared to efavirenz. Dolutegravir may lead to increases in long-term retention.

## Introduction

Globally, South Africa has the highest burden of HIV with approximately eight million people living with HIV (PLWH) [1,2]. As of 2022, South Africa has made substantial progress toward the UNAIDS 95–95-95 targets with 94% of the eight million PLWH knowing their HIV status, 79% of PLWH who know their status receiving antiretroviral therapy (ART), of whom 91% are virally suppressed [3,4]. Achieving these targets is critical to epidemic control but doing so will require identifying ways to improve retention in care [5,6]. Understanding retention in low- and middle-income countries, where the burden of HIV is greatest and where individuals have unique barriers to retention in care, is critical.

Following the uptake of dolutegravir in most high-income countries, South Africa’s National ART Guidelines were revised in October 2019 to recommend use of dolutegravir, an integrase-strand transfer inhibitor (INSTI), as part of first-line ART replacing efavirenz, a non-nucleoside reverse transcriptase inhibitor (NNRTI) [7]. These guidelines were updated in response to the World Health Organization’s (WHO) guidance and results from clinical trials demonstrating dolutegravir’s safety and efficacy with a more tolerable side effect profile [8–13]. While clinical trials have demonstrated dolutegravir’s high efficacy in terms of viral suppression and reduced toxicity, trials are not suitable for evaluating retention due to their intensive follow-up procedures and exclusion of clients with advanced disease or comorbidities. Therefore, robust observation studies are needed to evaluate the impact of dolutegravir on retention in HIV care. Observational studies have found that dolutegravir may improve retention and viral suppression compared to non-dolutegravir-based regimens [13–22]. However, many of these studies did not use efavirenz as the direct comparator to dolutegravir and/or included patients who were treatment experienced [14,16–18,21].

Furthermore, many prior observational studies were limited in their ability to identify causal effects due to residual confounding, misclassification and selection bias. Novel methods such as the target trial framework can help improve causal analyses using observational data [23]. The target trial framework is an approach to planning and designing analyses of observational data by emulating a hypothetical randomized controlled trial. Under this framework, researchers first design what an ideal randomized clinical trial would look like to address the causal question [23–25]. This involves explicitly stating all assumptions about treatment and study design, providing clear definitions of eligibility criteria and intervention, and defining a well specified zero time (i.e. when follow-up begins) to mitigate selection and immortal time bias (e.g. when treatment initiation occurs after zero time) [24,26,27]. Observational data is then used to explicitly emulate the protocol of the target trial when a trial is not feasible [28]. Prior work by our co-authors found no meaningful change in retention and suppression comparing dolutegravir to efavirenz; however, this analysis was limited to 6 months of follow-up and estimates were imprecise as the timing of this analysis was restricted to patients who initiated treatment within three months on either side of when the guideline change was implemented [29]. Therefore, evaluations of the impact of dolutegravir at 12 months and beyond utilizing novel analytic methods such as the target trial framework are needed.

Given the importance of understanding the impact of the policy change of replacing efavirenz with dolutegravir on retention and the challenges in estimating causal effects with observational data, we sought to estimate the effect of initiating a dolutegravir-based ART regimen vs. initiating an efavirenz-based ART regimen on 12- and 24-month retention and viral suppression utilizing the target trial framework.

## Methods

We emulated a hypothetical target trial using data from the Themba Lethu HIV Clinical Cohort. This clinical cohort has been described elsewhere [30]. Briefly, the clinic is a public-sector comprehensive HIV care and treatment clinic located at the Helen Joseph Hospital in Johannesburg, South Africa [30]. Since 2004, the clinic has initiated over 40,000 people onto ART based on South Africa’s National ART Guidelines for Adolescents and Adults [7,30]. Six months after treatment initiation patients receive viral load testing and then yearly thereafter [30]. Demographic, laboratory and clinical information is entered into TherapyEdge-HIV™, the clinic’s real-time data capturing system, at the time of the participant’s visit [30].

Prior to South Africa’s guideline revisions in 2019, participants were initiated onto one of the following first-line ART medications: tenofovir disoproxil fumarate/lamivudine/efavirenz or tenofovir disoproxil fumarate/emtricitabine/efavirenz [7]. After the guideline change, dolutegravir replaced efavirenz in these three-drug combinations for first-line ART [7].

### Target trial specification

Our target trial would enroll non-pregnant treatment-naïve patients eligible to start HIV treatment aged ≥16 years of age and randomize them to initiate either a dolutegravir-based regimen or an efavirenz-based regimen (Table 1). Zero time in this target trial would be the date each patient was randomized to their treatment strategy. Backbone regimens would be either tenofovir disoproxil fumarate/lamivudine or tenofovir disoproxil fumarate/emtricitabine. The primary outcome of the trial would be 12-month retention and viral suppression. The secondary outcome would be 24-month retention and viral suppression. Patients would be followed until the end of the study period at 24 months, loss to follow-up, or death. Retention would be defined as attending a visit at 12 and 24 months with a 3-month window on either side of each timepoint to allow for flexibility in study visit scheduling. Viral suppression would be defined as a viral load ≤50 copies/mL.

### Emulation of target trial and analytic approach

We emulated this target trial using data from the Themba Lethu HIV Clinical Cohort (Table 1). In addition to the eligibility criteria described in the target trial above, in the emulated trial patients also had to initiate first-line ART during the period 2019–2021. Zero time in this emulation was the date each patient met the eligibility criteria and initiated either a dolutegravir-based regimen or an efavirenz-based regimen. Patients were then followed until the earliest of (1) end of the study period, (2) loss to follow-up, (3) transfer or (4) death.

Our primary outcomes were retention at 12 months and viral suppression at 12 months, with secondary outcomes as 24-month versions of each. Retention was defined as whether a participant attended a visit at the clinic at 12 and 24 months within ±6 months of each endpoint allowing for variation in when participants scheduled their clinic appointments during the COVID-19 pandemic. Viral suppression was defined as having a viral load ≤50 copies/mL within ±6 months of each endpoint. This definition was used to allow for variation in the timing of when viral loads are conducted during the COVID-19 pandemic. The outcome of viral suppression was evaluated among those who had a viral load conducted which is also conditional upon being retained in care. Analyses evaluating 24-month outcomes were restricted to participants who initiated from 2019 to 2021 as participants who initiated in 2022 were not eligible for a full 24 months of follow-up.

**Figure 1.**
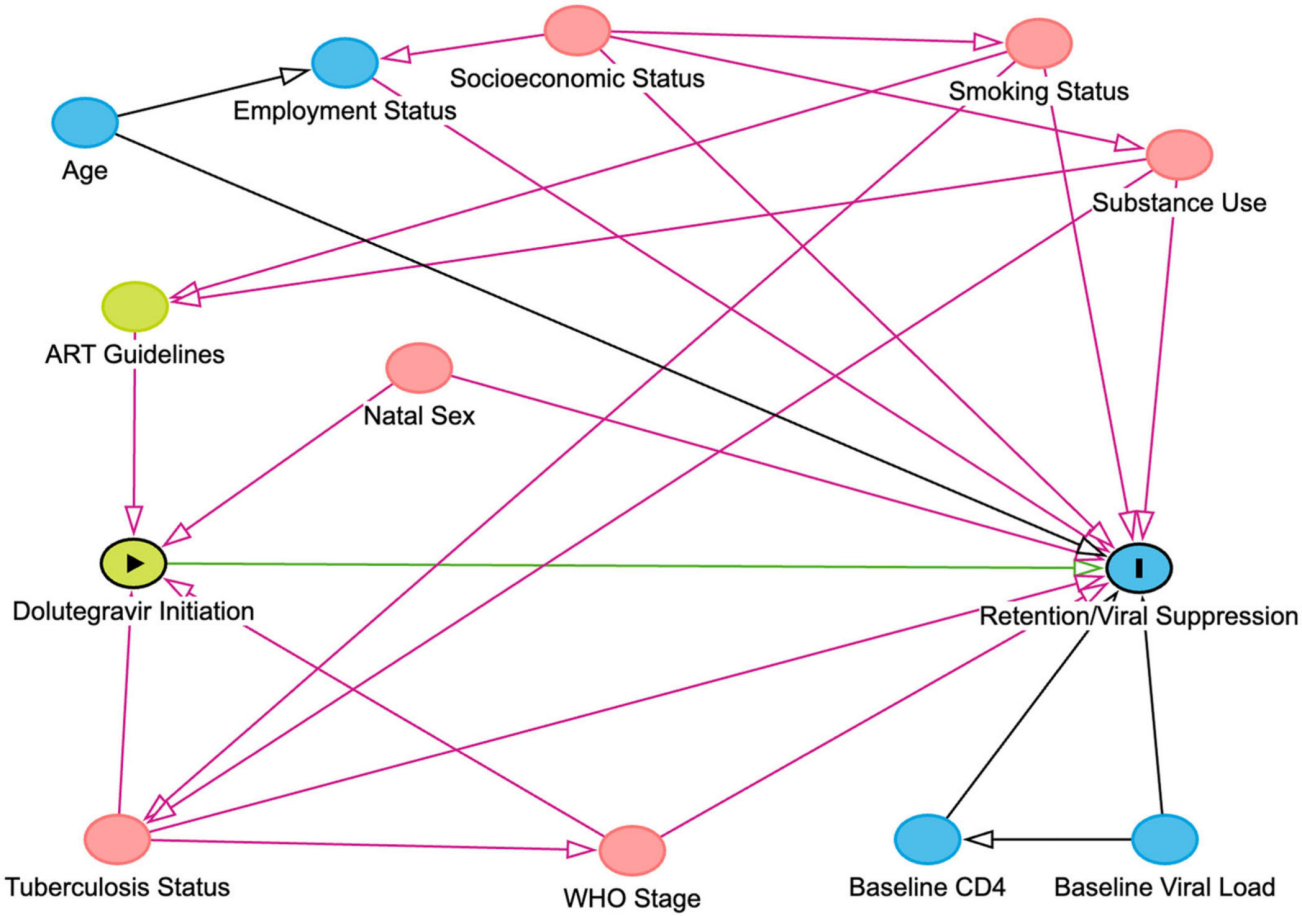
A directed acyclic graph of dolutegravir and retention/viral suppression.

Our analytic approach to creating balanced populations was to use inverse probability treatment weighting (IPTW) of marginal structural models. Specifically, we estimated the probability of treatment using multivariable logistic regression with exposure status as the outcome as a function of confounders of the relationship between the exposure (dolutegravir vs. efavirenz) and the outcomes of interest (retention and viral suppression at 12- and 24- months) [31–33]. A directed acyclic graph (DAG) was used to evaluate the potential causal relationships. Based on the DAG the following covariates were included in the model: natal sex, age, year of initiation, education level, employment status, tuberculosis, WHO stage, smoking and alcohol use (Figure 1). Similarly, we calculated censoring weights to reduce selection bias from loss to follow-up for the outcome of viral suppression [31]. Censoring weights for the retention were not calculated as loss to follow-up is part of the retention outcome. Based on the DAG, the following covariates were included for the censoring weights: natal sex, age, year of initiation, exposure status, education level, employment status, tuberculosis, WHO stage, smoking and alcohol use. At 24 months, probability of loss to follow-up also included whether the participant was retained at 12 months. Covariates with missing data were included in our treatment and censoring weights as we created a category to indicate missingness. Analyses stratified by sex utilized re-estimated weights where natal sex was excluded from the models. Analyses stratified by CD4 count utilized re-estimated weights where year of initiation was dichotomized to 2019 vs. 2020–2022 in the models with individuals who were missing a baseline CD4 count excluded from this analysis.

The data were then weighted by the inverse of the probability to create a pseudo-population in which measured confounders were balanced between treatment groups allowing us to evaluate the effect of initiating dolutegravir compared to efavirenz on retention and suppression, using pooled linear regression to estimate the causal risk difference [31]. Stabilized weights, truncated at the 99th percentile, were calculated by using the crude probability of exposure in the numerator instead of 1 [31]. Stabilized weights were used over unstabilized weights as they reduce the variance of the effect estimate that is calculated [31]. Robust confidence intervals (CIs) were obtained through non-parametric bootstrapping 100,000 times.

### Sensitivity analyses

To evaluate the robustness of our estimates, we conducted the following sensitivity analyses: (1) viral suppression was defined as a viral load ≤1000 copies/mL (e.g. a standard threshold for viral suppression), (2) restricted analyses of 12-month outcomes to participants who were eligible for a full 24 months of follow-up, (3) as having a viral load conducted is conditional upon being retained in care we assumed participants with a missing viral load were virally unsuppressed, (4) utilized stabilized weights truncated at the 95th percentile and (5) utilized unstabilized weights truncated at the 99th percentile.

### Ethics statement

Data from the Themba Lethu Clinic were collected for routine clinical purposes only and the study team had no direct contact with any of the participants. Use of the de-identified data was approved by the Human Research Ethics Committee of the University of the Witwatersrand (M140201) and the Institutional Review Board of Boston University (H-29768). An informed consent waived for analysis of the de-identified data was received from the Human Research Ethics Committee of the University of the Witwatersrand and the Institutional Review Board of Boston University.

## Results

### Baseline characteristics

In total, 2930 participants met trial emulation eligibility criteria and initiated ART treatment from 2019 to 2022. Of these, 1847 (63.0%) initiated a dolutegravir-based regimen and 1083 (37.0%) initiated an efavirenz-based regimen (Table 2). The overall median age was 45.1 years (interquartile range (IQR): 37.1, 53.0) with 52.3% (*n* = 1357) female. Most participants reported completing secondary school or greater and did not report any smoking or alcohol use (Table 2). When limited to 2019–2021 for the 24-month outcomes of the 2930 participants who met trial emulation criteria, 2297 initiated ART, of whom 1233 (53.7%) initiated a dolutegravir-based regimen and 1064 (46.3%) initiated an efavirenz-based regimen (Supplemental Table 1). Baseline demographics of this sub-population were similar to demographic characteristics of the overall cohort with a median age of 44.5 years (IQR: 36.5, 52.4) and 52.9% (*n* = 1215) female.

### Effect of dolutegravir on retention and viral suppression

**Figure 2.**
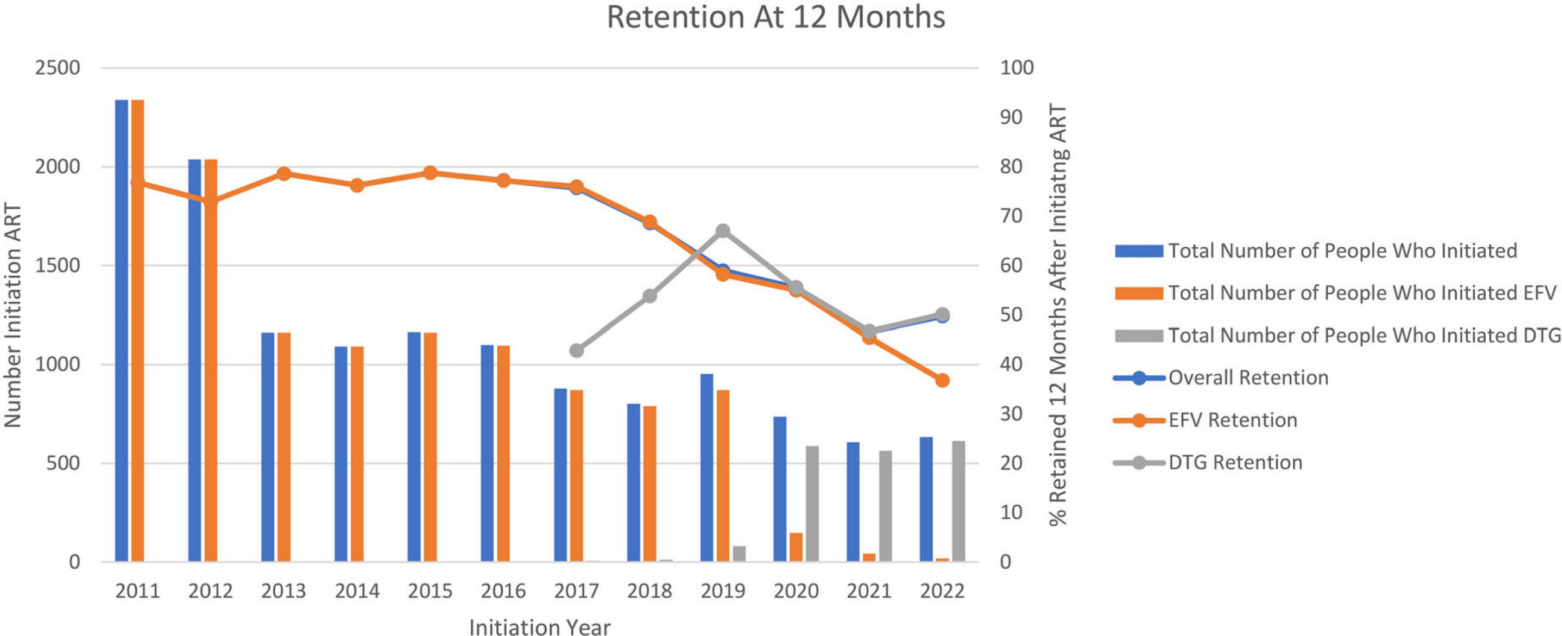
Retention in care at 12 months at Themba Lethu Clinic in Johannesburg, South Africa from 2011 to 2022.

Historically, 12-month retention at the clinic has been at or above 70.0% [34,35]. However, during the COVID-19 pandemic (e.g. 2019–2022) retention declined sharply due to the impact of lockdowns (Figure 2).

**Figure 3.**
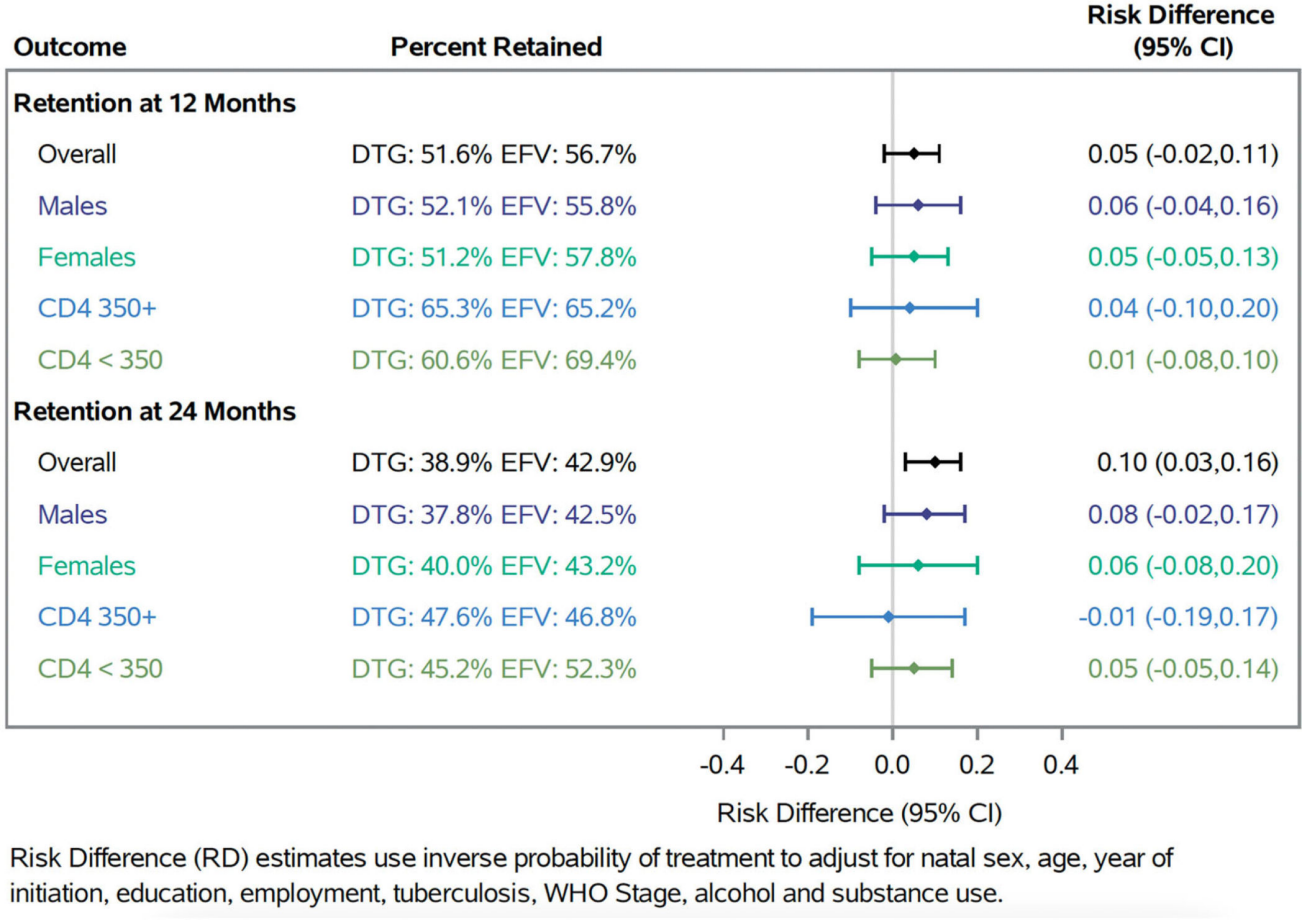
Forest plot of retention at 12 and 24 months at Themba Lethu Clinic in Johannesburg, South Africa from 2019 to 2022 overall and stratified by sex and CD4 count.

**Figure 4.**
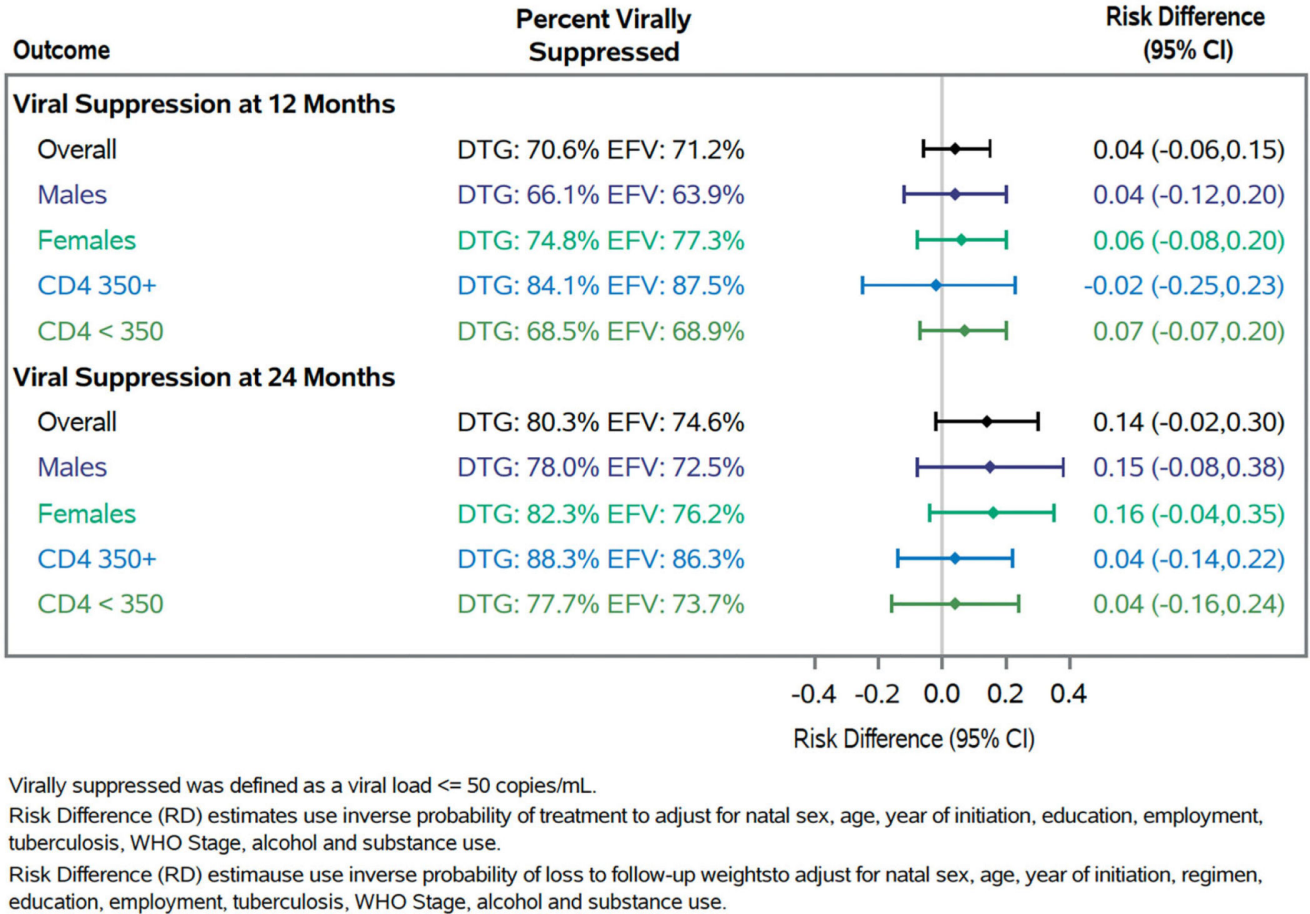
Forest plot of viral suppression at 12 and 24 months at Themba Lethu Clinic in Johannesburg, South Africa from 2019 to 2022 overall and stratified by sex and CD4 count.

At 12 months, 51.6% of patients who initiated a dolutegravir-based regimen were observed to be retained and of those retained, 70.6% were virally suppressed. Results were similar for those who initiated an efavirenz-based regimen with 56.7% retained and of those 71.2% were virally suppressed at 12 months. After weighting, we found a 5-percentage point (95% CI: −0.02, 0.11) increase in retention and a 4-percentage point (95% CI: −0.06, 0.16) increase in viral suppression among those retained for individuals initiating a dolutegravir-based ART regimen compared to those initiating an efavirenz-based ART regimen at 12 months (Figures 3 and 4). At 24 months, among participants who initiated a dolutegravir-based regimen, 38.9% were retained and among those 80.3% were virally suppressed. Comparatively, among participants who initiated an efavirenz-based regimen, 42.9% were retained and among those 74.6% were virally suppressed at 24 months. At 24 months after weighting, we found a 10-percentage point (95% CI: 0.03, 0.16) increase in retention and a 14-percentage point (95% CI: −0.02, 0.30) increase in viral suppression. When stratified by sex, we found similar results at 12 and 24 months. When stratified by CD4 count, effects were slightly attenuated for both strata but less precise.

### Sensitivity analyses

We conducted five separate sensitivity analyses. Estimates from the first three sensitivity analyses were less precise, but consistent for 12-month viral suppression with risk differences ranging from 3% to 8% at 12 months. At 24 months, these estimates were less precise and slightly attenuated with the risk difference ranging from −0.6% to 5% (Supplemental Tables 2 and 3). Fourth, when we re-ran all analyses using the stabilized weight truncated at the 95th percentile, we found no difference in retention at 12 months with a risk difference of −0.003% (95% CI: −5%, 5%) and a slightly attenuated risk difference of 7% (95% CI: 1%, 13%) at 24 months. Effect estimates for viral suppression were consistent with a risk difference of 3% (95% CI: −5%, 11%) at 12 months and 15% (95% CI: 4%, 25%) at 24 months (Supplemental Table 4). Lastly, when we utilized unstabilized weight truncated at the 99th percentile, estimates were less precise but consistent at 12 and 24 months for both retention and suppression (Supplemental Table 5). Overall, results from our sensitivity analyses did not substantively change the conclusions our findings.

## Discussion

Our findings suggest that any effect between a 2% decrease in retention to an 11% increase in retention over the first 12 months of initiating a dolutegravir-based regimen compared to an efavirenz-based regimen initiation is compatible with the data in this study, with differences closer to 5% being most compatible. At 24 months, our results suggest that any effect between a 3% and a 16% increase in 24-month retention is compatible with the data in this study. Based on these results, over the long term, dolutegravir may lead to a moderate improvement in retention and a small improvement viral suppression when compared to efavirenz with no notable differences when stratified by sex or CD4 count. In an earlier study using data from this cohort, we found no difference in retention and viral suppression at 6 months [29]. However, this is likely due to that fact that this analysis was limited by a small sample size and short follow-up time. We note that even a null effect could be considered a positive result as dolutegravir has fewer side effects and improved tolerability when compared to efavirenz [10,11,13,22]. Some side effects from ART only become apparent over the long-term, necessitating analyses such as this one evaluating these outcomes at 12 and 24 months.

Findings from this current analysis are in line with results from clinical trials and other observational studies that have found improvement in retention and are likely due improved tolerability (e.g. less severe side effects and adverse events) of dolutegravir when compared to efavirenz [9,11,13,14]. The improved tolerability may also explain why there were no differences when stratified by sex or CD4 count. While clinical trials found no difference in viral suppression at 96 weeks and beyond between those who initiated a dolutegravir-based regimen compared to an efavirenz-based regimen, some observational studies have found improvements in viral suppression at 12 months [10,11,14,15,22]. A retrospective cohort study that was conducted among clinics in eThekwini, South Africa found better retention and viral suppression at 12 months for those who initiated a dolutegravir-based regimen [14]. This indicates that not only is it important to continue evaluating the effects of these ART medications over the long-term, especially as some ART side effects become more apparent at 18 and 24 months, but the necessity of observational effectiveness studies as clinical trials are often not representative of real-world clinical settings. These results, indicating a 10-percentage point improvement in 24-month retention, are potentially clinically meaningful and could support South Africa in reaching the UNAIDS 95–95-95 targets. It is likely that a five-percentage point change in retention and viral suppression is a meaningful benefit for patients and health systems as the UNAIDS transitioned from the 90–90-90 targets to the 95–95-95 targets which suggests a five-percentage point increase is important [3]. However, even small improvements in long-term retention extend beyond individual patient health, carrying important implications for public health by preventing onward transmission and achieving an HIV-free generation [3,5,6].

Our analyses took place during the COVID-19 pandemic (e.g. 2019–2022). During this period, South Africa implemented lockdowns and travel restrictions that affected access to the clinic, changed dispensing patterns for ART, increased the number of self-transfers, and overall increased morbidity and mortality related to COVID-19. Legislation restricted travel and prevented patients from accessing public HIV clinics [36]. The Themba Lethu Clinic allowed multi-month (up to 3 months) ART dispensing reducing the frequency of visits, many patients transferred to other clinics as they relocated out of the province and/or country due to COVID-19 pandemic related job losses, and sometimes visits/corresponding lab results that did take place were not recorded due to resource-constraints during this time period. We suspect that a combination of these factors explains why (1) our observed retention rates at 12 and 24 months are lower than the clinic’s historic retention of around 70.0% and (2) a number of participants had missing viral loads [34,35]. Considering this, the retention numbers we report should be considered ‘retained’ at site. Several studies conducted prior to the COVID-19 pandemic in sub-Saharan Africa, including in South Africa, have demonstrated that many patients self-transfer and the percent retained is likely to be much higher at the national level (e.g. >70%) [35,37,38]. A recent study evaluating 12-month retention among patients who initiated ART from 2018 to 2022 across 18 facilities in Gauteng, Mpumalanga and KwaZulu Natal provinces in South Africa also found similar rates of 12-month retention with 51.8% of all patients, regardless of ART regimen, retained in care [39]. When stratified by year of initiation and province, retention in care at 12 months was lower ranging from 41.0% to 54.2% during the COVID-19 pandemic [39]. Future studies are needed to evaluate how retention may change in the coming years following the COVID-19 pandemic, as current trends remain unclear.

Furthermore, having a viral load is conditional upon being retained in care so those with a completed viral load test may be more likely to be virally suppressed compared to those without a viral load test as being retained in care increases the chances patients are adherent. However, our sensitivity analysis where we assumed that participants who were missing a viral load were virally unsuppressed did not substantially change the conclusions of our findings. Our observational emulation had two key differences when compared to the target trial. The first difference was that for the 24-month outcomes we had to subset our cohort of patients initiating ART from 2019 to 2022 to 2019–2021 as those who initiated in 2022 were not eligible for a full 24 months of follow-up. The second difference was with the definitions of retention and viral suppression. The target trial utilized a 3-month window on either side of each timepoint whereas our observational emulation utilized a 6-month window on either side. The use of a 6-month window was done to account for the changes to visit scheduling and ART dispensing procedures at the clinic during the pandemic. Inverse probability treatment weighting relies on untestable assumptions of conditional exchangeability, consistency and correct model specification [31]. Additionally, our stratified CD4 analyses excluded individuals with missing baseline CD4 data. This could potentially bias our results toward the null; however, this bias is likely to be to non-differential with respect to treatment strategy as both treatment groups had equal amounts of missing data for baseline CD4 count. Lastly, our study was conducted at a single, urban clinic in Johannesburg. Patients receiving care at the at the clinic may be at higher risk of treatment failure compared to participants receiving care at other ART clinics in South Africa as the clinic has, in recent years, been a referral clinic for patients with adherence issues and complex disease. Therefore, our findings may not be applicable to other settings across South Africa.

## Conclusions

Results from our study indicate that over 24 months patients initiating a dolutegravir-based regimen are more likely to be retained in care and virally suppressed when compared to patients initiating an efavirenz-based regimen. This improvement in retention and viral suppression are possibly because of dolutegravir’s improved tolerability compared to efavirenz, resulting in fewer treatment discontinuations. Evaluating the impact of this policy change on treatment outcomes at 12 months and beyond is essential to ensure South Africa is on track to achieve the 95–95-95 targets by 2030. It is important to note that while a dolutegravir-based regimen may be better tolerated and improve HIV treatment outcomes, there is growing evidence that it may lead to increased weight gain and subsequently increase a patient’s risk for diabetes and hypertension [40,41]. Future work should focus on evaluating the long-term risk of weight gain, hypertension and diabetes among these patients as these may also impact adherence and retention in care over a patient’s lifespan.

## Supplementary Material

Supp 1

Supplemental data for this article can be accessed online at https://doi.org/10.1080/23744235.2025.2557628.

## Figures and Tables

**Table 1.** Differences and similarities between the target trial and the observational emulation of the effect of dolutegravir vs. efavirenz on retention and viral suppression as 12 and 24 months.

Component	Target trial	Observational emulation
Eligibility criteria	Participants newly initiating first-line ART, aged 16+ and not pregnant at treatment initiation	Participants newly initiating first-line ART from 2019 to 2022, aged 16+ and not pregnant at treatment initiation
Treatment strategies	Initiate on dolutegravir vs. initiate on efavirenz	Same criteria
Treatment assignment mechanism	Randomization to ensure exchangeability of the treatment groups	Adjust for: natal sex, age, year of initiation, education level, employment status, tuberculosis, WHO stage, smoking and alcohol use
Start of follow-up	Follow-up starts at randomization	Follow-up begins the day eligibility criteria are met
End of follow-up	Ends at the last study visit when the last outcome measure is taken	Ends after 24 months
Outcomes	Viral suppression and retention at 12 and 24 months with a 3-month window on either side	Viral suppression and Retention at 12 and 24 months with a 6-month window on either side
Causal contrast	Intention-to-treat effect	Same criteria

**Table 2.** Baseline demographics of individuals initiating ART at the Themba Lethu HIV Clinic in Johannesburg, South Africa from 2019 to 2022 by ART regimen (*N*= 2930).

	Dolutegravir-based regimen (*n*= 1847)	Efavirenz-based regimen (*n*= 1083)	Overall (*N*= 2930)
Age (years)	45.5 (37.7, 53.7)	44.2 (35.9, 51.9)	45.1 (37.1, 53.0)
Sex			
Female	952 (51.5)	585 (54.0)	1537 (52.3)
Male	895 (48.5)	498 (46.0)	1393 (47.5)
Education level^a^			
Less than primary school	1 (0.1)	4 (0.5)	5 (0.3)
Primary school	113 (13.9)	68 (9.0)	181 (11.5)
Secondary school and above	698 (86.0)	685 (90.5)	1383 (88.1)
Missing	1035 (56.0)	326 (30.1)	1361 (46.5)
Alcohol use^a^			
No	984 (81.0)	696 (82.5)	1680 (81.6)
Prior history	6 (0.5)	5 (0.6)	11 (0.5)
Yes	225 (18.5)	143 (16.9)	368 (17.9)
Missing	632 (34.2)	239 (22.1)	871 (29.7)
Smoking^a^			
No	1007 (83.4)	708 (84.8)	1715 (83.9)
Prior history	2 (0.2)	2 (0.2)	4 (0.2)
Yes	199 (16.5)	125 (15.0)	324 (15.9)
Missing	639 (34.6)	248 (22.9)	887 (30.3)
Tuberculosis	138 (7.5)	61 (5.6)	199 (6.8)
CD4 count (mean (SD))	242.9 (1202.2)	273.4 (298.5)	254.1 (975.3)
CD4 count (median [IQR])	132.0 [34.0–316.0]	178.0 [48.0–397.0]	142.0 [36.0–337.0]
CD4 count^a^			
<350	957 (78.7)	496 (71.2)	1453 (76.0)
≥350	259 (21.3)	201 (28.8)	460 (24.0)
Missing	631 (34.2)	386 (35.6)	1017 (34.7)
WHO stage			
Stages 1 and 2	1541 (83.4)	926 (85.5)	2467 (84.2)
Stages 3 and 4	306 (16.6)	157 (14.5)	463 (15.8)

a Percentages for missing are based off the total *n* for each group, but the percentages for the true values are based off the total of non-missing values.
