# Supplementary material for: Initiation of dolutegravir vs. efavirenz on 12- and 24-month retention and viral suppression: a target trial emulation
Source: Infect Dis (Lond). Author manuscript; Available in PMC 2025 Nov 13. (PMC12606652; doi:10.1080/23744235.2025.2557628)
Supplement: Supp 1 [file NIHMS2113464-supplement-Supp_1.docx]

**Supplemental Table 1.** Baseline demographics of individuals initiating ART from 2019-2021 by ART regimen (N = 2297)

|  | Dolutegravir-based Regimen  (n = 1233) | Efavirenz-based Regimen  (n = 1064) | Overall  (N =2297) |
| --- | --- | --- | --- |
| **Age** (Years) | 44.8 (37.2, 53.0) | 44.2 (35.8, 51.9) | 44.5 (36.5, 52.4) |
| **Sex** | | | |
| *Female* | 638 (51.7) | 577 (54.2) | 1215 (52.9) |
| *Male* | 595 (48.3) | 487 (45.8) | 1082 (47.1) |
| **Education Level**^1^ | | | |
| *Illiterate* | 1 (0.2) | 4 (0.5) | 5 (0.4) |
| *Primary School* | 51 (9.1) | 65 (8.7) | 116 (8.9) |
| *Secondary School and Above* | 508 (90.7) | 681 (90.8) | 1189 (90.7) |
| *Missing* | 673 (54.6) | 314 (29.5) | 987 (43.0) |
| **Alcohol Use**^1^ | | | |
| *No* | 668 (80.0) | 691 (82.7) | 1359 (81.3) |
| *Prior History* | 4 (0.5) | 5 (0.6) | 9 (0.5) |
| *Yes* | 163 (19.5) | 140 (16.7) | 303 (18.1) |
| *Missing* | 398 (32.2) | 228 (21.4) | 626 (27.3) |
| **Smoking**^1^ | | | |
| *No* | 692 (83.7) | 700 (81.7) | 1392 (84.2) |
| *Prior History* | 1 (0.1) | 2 (0.2) | 3 (0.2) |
| *Yes* | 134 (16.2) | 125 (14.6) | 259 (15.7) |
| *Missing* | 406 (32.9) | 237 (22.3) | 643 (28.0) |
| **Tuberculosis** | 122 (9.9) | 60 (5.6) | 182 (7.9) |
| **CD4 Count** (Mean (SD)) | 254.0 (1470.1) | 275.4 (200) | 263.9 (1097.3) |
| **CD4 Count** (Median [IQR]) | 123.0 [31.0-301.0] | 184.0 [48.0-404.0] | 141.0 [35.5-342.0] |
| **CD4 Count**^1^ | | | |
| *< 350* | 639 (79.6) | 488 (70.8) | 1127 (75.5) |
| *> 350* | 164 (20.4) | 488 (29.2) | 365 (24.5) |
| *Missing* | 430 (34.9) | 375 (35.2) | 805 (35.0) |
| **WHO Stage** | | | |
| *Stages 1 and 2* | 1002 (81.3) | 913 (85.8) | 1915 (83.4) |
| *Stages 3 and 4* | 231 (18.7) | 151 (14.2) | 382 (16.6) |

^1^Percentages for missing are based off the total n for each group, but the percentages for the true values are based off the total of non-missing values

**Supplemental Table 2.** Sensitivity Analysis 1 and 2: Risk Difference and 95% Confidence Interval for Viral Suppression at 12- and 24-Months

|  | **Dolutegravir** | | **Efavirenz** | | ***Risk Difference (95% CI)*** | |
| --- | --- | --- | --- | --- | --- | --- |
|  | *12-Months* | *24-Months* | *12-Months* | *24-Months* | *12-Months* | *24-Months* |
| Virally Suppressed^1^ | 645 (92.9) | 344 (95.6) | 407 (88.7) | 361 (90.7) | 0.08 (0.003, 0.16) | 0.05 (-0.06, 0.15) |
| Virally Suppressed^2^ | 490 (26.5) | 289 (23.4) | 327 (30.2) | 297 (27.9) | 0.03 (-0.03, 0.09) | 0.03 (-0.03, 0.08) |
| Virally Suppressed^3^ | 645 (34.9) | 344 (27.9) | 407 (37.6) | 361 (33.9) | 0.05 (-0.01, 0.11) | -0.006 (-0.08, 0.06) |

^1^Virally suppressed was defined as a viral load < 1000 copies/mL; Percent virally suppressed is based on those with a documented viral load (Dolutegravir n = 694 at 12 months and n = 360 at 24 months; Efavirenz n = 459 at 12 months and n = 398 at 24 months)

^2^Virally suppressed was defined as a viral load < 50 copies/mL, if an individual was missing a viral load they were assumed to be not virally suppressed

^3^Virally suppressed was defined as a viral load < 1000 copies/mL, if an individual was missing a viral load they were assumed to be not virally suppressed

**Supplemental Table 3.** Sensitivity Analysis 3: Risk Difference and 95% Confidence Interval for Retention and Viral Suppression at 12- Restricted to Individuals with 24-Months of Follow-up Available

|  | **Dolutegravir**  (n = 1233) | **Efavirenz**  (n = 1064) | ***Risk Difference (95% CI)*** |
| --- | --- | --- | --- |
| Retained | 645 (52.3) | 609 (57.2) | 0.05 (-0.02, 0.11) |
| Virally Suppressed^1^ | 358 (72.5) | 325 (71.3) | 0.07 (-0.04, 0.18) |

^1^Virally suppressed was defined as a viral load < 50 copies/mL; Percent virally suppressed is based those with a documented viral load (Dolutegravir n = 494 at 12 months; Efavirenz n = 456 at 12 months)

**Supplemental Table 4.** Sensitivity Analysis 4: Risk Difference and 95% Confidence Interval for Retention and Viral Suppression at 12- and 24-Months with Stabilized Weights Truncated at the 95^th^ Percentile

|  | ***Risk Difference (95% CI)*** | |
| --- | --- | --- |
|  | *12-Months* | *24-Months* |
| Retained | -0.00003 (-0.05, 0.05) | 0.07 (0.01, 0.13) |
| Virally Suppressed^1^ | 0.03 (-0.05, 0.11) | 0.15 (0.04, 0.25) |
| Virally Suppressed^2^ | 0.07 (0.01, 0.12) | 0.06 (-0.01, 0.13) |
| Virally Suppressed^3^ | 0.02 (-0.03, 0.06) | 0.06 (0.008, 0.11) |
| Virally Suppressed^4^ | 0.03 (-0.02, 0.08) | 0.03 (-0.02, 0.09) |

^1^Virally suppressed was defined as a viral load < 50 copies/mL

^2^Virally suppressed was defined as a viral load < 1000 copies/mL

^3^Virally suppressed was defined as a viral load < 50 copies/mL, if an individual was missing a viral load they were assumed to be not virally suppressed

^4^Virally suppressed was defined as a viral load < 1000 copies/mL, if an individual was missing a viral load they were assumed to be not virally suppressed

**Supplemental Table 5.** Sensitivity Analysis 5: Risk Difference and 95% Confidence Interval for Retention and Viral Suppression at 12- and 24-Months Truncated at 99^th^ Percentile with Unstabilized Weight

|  | ***Risk Difference (95% CI)*** | |
| --- | --- | --- |
|  | *12-Months* | *24-Months* |
| Retained | 0.05 (-0.02, 0.11) | 0.09 (0.04, 0.16) |
| Virally Suppressed^1^ | 0.04 (-0.06, 0.15) | 0.13 (-0.02, 0.28) |
| Virally Suppressed^2^ | 0.08 (0.0006, 0.16) | 0.04 (-0.06, 0.14) |
| Virally Suppressed^3^ | 0.03 (-0.03, 0.09) | 0.03 (-0.04, 0.09) |
| Virally Suppressed^4^ | 0.05 (-0.01, 0.11) | -0.01 (-0.09, 0.07) |

^1^Virally suppressed was defined as a viral load < 50 copies/mL

^2^Virally suppressed was defined as a viral load < 1000 copies/mL

^3^Virally suppressed was defined as a viral load < 50 copies/mL, if an individual was missing a viral load they were assumed to be not virally suppressed

^4^Virally suppressed was defined as a viral load < 1000 copies/mL, if an individual was missing a viral load they were assumed to be not virally suppressed
